# Bispecific protein targets prostate cancer

**DOI:** 10.18632/oncotarget.17372

**Published:** 2017-04-23

**Authors:** Antony W. Burgess

**Affiliations:** Walter and Eliza Hall Institute, Parkville, Australia

**Keywords:** PSMA, polyIC, immunotherapy, dsRNA, prostate cancer

Prostate cancer is the second most common cancer in men worldwide, and the eighth leading cause of cancer-related death. Globally, there are approximately 1,100,000 new cases and 300,000 mortalities every year, comprising nearly 4 percent of all cancer deaths. It is estimated that 1 in every 7 men will be diagnosed with the disease during his lifetime[1].

The most dangerous stage of prostate cancer is bone metastasis, where the cancer spreads and forms new solid tumors in the bone. These are the tumor cells which need to be destroyed.

When the cancer has spread metastasized, the 5-year survival rate drops to 28% [1]; Although androgen deprivation therapy offers improved survival for some patients [2] there are considerable risks associated with these treatments. As yet there are no effective treatments for patients with advanced-stage prostate cancer.

The current issue of Oncotarget includes an article [3] describing the production of a bispecific fusion protein capable of binding to and the specific delivery of a cytotoxic dsRNA to metastatic prostate cancer cells. In previous studies Levitzki has shown that a complex chemical vector (LPEI-PEG-peptide-poly-IC) can be used to target cancer cells which over express the EGFR[4]. The new fusion protein is more precisely engineered with a single chain antibody fragment to target the surface of cells expressing the prostate-specific membrane antigen (PSMA), a protein domain to bind and carry the dsRNA and a positively charged linker which disrupts the endosomal membrane and allows the dsRNA to enter the cytoplasm of the cancer cells. As well as inducing cell death the cytoplasmic dsRNA stimulates a cytokine storm capable of attracting and activating the immune system to kill even more tumor cells.

There is considerable interest in activating the immune system to target prostate specific antigens (e.g. Provenge®[5] :prostatic acid phosphatase; (PROSTVAC[6]: prostate specific antigen, PSA; and BAY2010112[7]: a CD3 and PMSA bispecific monoclonal antibody). These studies are already in the clinic and are demonstrating that activation of the immune system is necessary for anti-tumor responses. Although it may be possible to cause significant radiation based killing of prostate cancers with radiolabeled antibodies [8], the delivery of sufficient tumor radiation is expensive and can be associated with significant logistic/safety issues in many hospitals.

Levitzki's team had to design the preparation and properties of the fusion proteins with considerable care. Although the dsRB domains can bind poly-IC, in the initial production these domains bind endogenous nucleic acids. They removed any endogenous nucleic acids by denaturing the fusion protein and refolding it so that both the dsRB and PSMA binding domains were functional. This fusion protein is more homogeneous than previous chemical polymers designed to deliver poly-IC. This protein vector is only successful because in between the two functional domains, there is an Arg9 linker, which is cleaved by endosomal proteases, releasing peptide, which disrupts the endosomal membrane and allows entry of the poly-IC into the cytoplasm of the cancer cells. The delivery of the poly-IC via the dsRB domain and PSMA targeting increases its effectiveness, so lower doses can be used for killing the tumor cells and there should be less non-specific toxicity.

This specific targeting, improved delivery and the bystander effects achieved by this bispecific fusion protein have yielded a new approach to a therapeutic to help patients with advanced prostate cancer. Given the long lead times and limited efficacy of the current anti-prostate cancer vaccine trials, this bispecific protein vector should provide an attractive alternative approach for improving the treatment of metastatic castration-resistant prostate cancer.
